# The Cases of Collins and Whitmarsh

**Published:** 1898-11-05

**Authors:** 


The Hospital

A Journal of

The Medical Sciences and Hospital Administration.

Edited by Sir Henry Burdett, K.C.B., and Solomon C. Smith, M.D., M.R.C.P.

Vol. XXV.—No. 632.] [Nov. 5, 1898.

### The Cases of Collins and Whitmarsh.

Considerable surprise has been expressed in many quarters at the illustration of "the glorious uncertainty of the law" which has recently been furnished by the different verdicts returned by juries in the cases of the two medical practitioners, Collins and Whitmarsh, who, within a short period of time, have both been tried for causing death in the course of an attempt to procure abortion. Collins, it will be remembered, was tried before Mr. Justice Grantham, convicted of manslaughter, and sentenced to penal servitude. Whitmarsh was first tried by a jury the members of which were discharged because unable to agree, and afterwards, before Mr. Justice Bigham, by a jury who convicted him of murder, so that he was sentenced to death; the jury at the same time strongly recommending him to the mercy of the Crown. The explanation of the difference is to be found in the absence of a Court of Criminal Appeal, and in the different opinions entertained by different judges upon a question on which the verdict of the jury was made to turn. It is settled law that any person who commits a felony, and who, in course of his felonious act, causes death, is guilty of murder; and it is not settled law that any surrounding circumstances can reduce the crime to manslaughter. Some judges are of opinion that they can, and others hold equally strongly that they cannot. Mr. Justice Grantham entertains the former view, and he instructed the jury in the case of Collins that it was within their competency to return a verdict of manslaughter. Mr. Justice Bigham entertains the latter view, and he instructed the jury in the case of Whitmarsh that the offence was murder or nothing, or that it could not be reduced to manslaughter unless they acquitted the prisoner of the felony and only found him guilty of causing death by criminal negligence. This was impossible in the face of the evidence, and the verdict given was in accordance with the ruling of the Bench. The difference is at first sight puzzling; but the practical importance attaching to it is much diminished by the revising power of the Crown, which, as was expected, has already been so exercised in favour of Whitmarsh as to reduce his punishment to something like that inflicted upon Collins. At the same time, it would be very desirable, if possible, to diminish the risks of so great a diversity between the verdicts and sentences in two cases bearing close resemblance to each other. No one can wish to see our judges all turned out with minds of precisely the same pattern, and all taking the same view of any uncertainties in the laws which they are called upon to administer; but, if we had a Court of Criminal Appeal, the necessity for which has often been urged by eminent lawyers, such a point as that which we have mentioned would be argued, and cleared up once for all, with the manifestly desirable result of increasing the uniformity of criminal procedure.

The different views to which we have called attention are easily explicable if we look at the language which has been used by jurists with regard to the distinction between murder and manslaughter. Blackstone says that the law would presume malice in every case of homicide, and would regard it as murder, "unless it were excused on account of accident or self-preservation, or alleviated into manslaughter by being the involuntary consequence of some act *not strictly lawful*." The last words can hardly be held to include the commission of a felony, or at least would not be so held by the majority of people. They rather point to some slight and casual offence, such as an assault of ordinary character, a trespass, or a blow struck in heat and under the immediate influence of provocation. We have no doubt that many will find themselves in agreement with Mr. Justice Bigham, and will consider that a death caused by the deliberate commission of a felony, more especially of a felony the motives for which were purely and entirely sordid and mercenary, cannot be resolved into a small matter, to be adequately punished even by a term of penal servitude. Still, we must not lose sight of the fact that the milder interpretation may, on the whole, be more calculated than the other to afford a much-needed protection to public morality and safety. The primary offence is one about which it is useless to be squeamish, or to shut our eyes to facts; and all who refuse to do this must be aware that the crime of procuring abortion has of late years been greatly on the increase, and that persons who ought to know better have come to look upon it with a certain degree of laxity. The lives of vast numbers of unborn children, and of many prospective mothers, are annually sacrificed for the sake of self-indulgence; and the increasing prevalence of a belief that the process is comparatively easy has removed at least one restraint from some to whom maternity would be shame. An agreement among the judges to regard the offence as possibly falling under the head of manslaughter would probably be extremely helpful in procuring convictions; and every conviction would serve not only to diminish the number of offenders, but also to educate the public mind with regard to the true heinousness of the offence.

